# A Novel Triple-Cell Two-Dimensional Model to Study Immune-Vascular Interplay in Atherosclerosis

**DOI:** 10.3389/fimmu.2019.00849

**Published:** 2019-04-24

**Authors:** Jonathan Noonan, Gianluca Grassia, Neil MacRitchie, Paul Garside, Tomasz J. Guzik, Angela C. Bradshaw, Pasquale Maffia

**Affiliations:** ^1^Centre for Immunobiology, College of Medical, Veterinary and Life Sciences, Institute of Infection, Immunity and Inflammation, University of Glasgow, Glasgow, United Kingdom; ^2^College of Medical, Veterinary and Life Sciences, Institute of Cardiovascular and Medical Sciences, University of Glasgow, Glasgow, United Kingdom; ^3^Department of Internal and Agricultural Medicine, Jagiellonian University College of Medicine, Kraków, Poland; ^4^Department of Pharmacy, University of Naples Federico II, Naples, Italy

**Keywords:** 2D model, atherosclerosis, endothelial cells (ECs), macrophages, smooth muscle cells (SMCs)

## Abstract

Atherosclerosis is a complex inflammatory pathology underpinning cardiovascular diseases (CVD), which are the leading cause of death worldwide. The interplay between vascular stromal cells and immune cells is fundamental to the progression and outcome of atherosclerotic disease, however, the majority of *in vitro* studies do not consider the implications of these interactions and predominantly use mono-culture approaches. Here we present a simple and robust methodology involving the co-culture of vascular endothelial (ECs) and smooth muscle cells (SMCs) alongside an inflammatory compartment, in our study containing THP-1 macrophages, for studying these complex interactions. Using this approach, we demonstrate that the interaction between vascular stromal and immune cells produces unique cellular phenotypes and soluble mediator profiles not observed in double-cell 2D cultures. Our results highlight the importance of cellular communication and support the growing idea that *in vitro* research must evolve from mono-culture systems to provide data more representative of the multi-cellular environment found *in vivo*. The methodology presented, in comparison with established approaches, has the advantage of being technically simple whilst enabling the isolation of pure populations of ECs, SMCs and immune cells directly from the co-culture without cell sorting. The approach described within would be applicable to those studying mechanisms of vascular inflammation, particularly in relation to understanding the impact cellular interaction has on the cumulative immune-vascular response to atherogenic or inflammatory stimuli.

## Introduction

Despite global efforts to prevent and treat cardiovascular diseases, myocardial infarction and stroke remain leading causes of mortality worldwide (1, 2). The common pathological link between these clinical outcomes is atherosclerosis, a progressive inflammatory disease of the arteries (3–5). The predominant cellular components of arteries are vascular endothelial (ECs) and smooth muscle cells (SMCs), both of which play key roles in the development of atherosclerosis. EC dysfunction develops in response to perturbations of normal blood flow combined with risk factors such as hypercholesterolemia (6, 7). In parallel, SMCs undergo phenotypic changes, paradoxically contributing to both the structural stabilization of atherosclerotic lesions but at the same time enhancing local inflammation (8). Equally, immune responses play a key role in atherosclerosis. The recruitment of monocytes by activated ECs and their differentiation into inflammatory macrophages in the tissue represents a hallmark of the disease (9, 10). As pathology progresses the contribution of immune cells to atherosclerotic plaque composition increases, with the formation of arterial tertiary lymphoid organs (11–13). Increasing understanding of immune cell heterogeneity in atherosclerotic lesions, using single cell RNA sequencing and Time of Flight Mass Cytometry (14, 15) supports the need to study interactions of defined subsets of immune cells with endothelial and vascular smooth muscle cells in detail. However, this is hard to address *in vivo*. Given the huge complexity of atherosclerotic disease and the interplay between stromal and immune cells, the use of co-culture systems has been identified as an important and advantageous approach to *in vitro* research (16). Several vascular-immune co-culture models have been published to date (16–21). However, whilst these models are certainly useful, they often suffer from being technically demanding, for example requiring custom culture scaffolds or perfusion equipment. Given this limitation, we aimed to develop a simplified *in vitro* co-culture approach for studying the cumulative response of key vascular cells alongside a flexible immune/inflammatory compartment.

Here we describe a triple-compartment cell culture model comprising of ECs, SMCs and an immune/inflammatory component, which in this study consisted of THP-1 macrophages. This model has the advantage of being technically simple whilst allowing for the independent isolation of each cellular compartment for downstream analysis without cell sorting. Moreover, this model could be further customized and enhanced via the introduction of more complex or alternative immune/inflammatory components.

## Materials and Methods

### Cell Culture

Human coronary artery endothelial cells (ECs) and human coronary artery smooth muscle cells (SMCs) were purchased from PromoCell (Heidelberg, Germany; lot numbers 4071602 and 4082801.2, respectively), and used between passage six and nine for all experiments. All vascular cell culture media used was purchased from PromoCell and are detailed in Table 1. Monocultures of all vascular cells were maintained in 75 cm^2^ tissue culture treated vented flasks (Corning, NY, USA) in a 37°C and 5% CO^2^ environment in the appropriate media.

**Table 1:** Cell culture media and supplements.

**Cells in culture**	**Product name (supplement components)**
Endothelial cell monoculture	Endothelial cell growth medium (Fetal calf serum (0.02 mL/mL), Endothelial Cell Growth Supplement/ECGS (0.004 mL/mL), human recombinant epidermal growth factor (0.1 ng/mL), human recombinant basic fibroblast growth factor (1 ng/mL), heparin (90 μg/mL), hydrocortisone (1 μg/mL), penicillin, streptomycin).
Smooth muscle cell monoculture	Smooth muscle cell growth medium 2 (Fetal calf serum (0.05 mL/mL), human recombinant epidermal growth factor (0.5 ng/mL), human recombinant fibroblast growth factor (2 ng/mL), human recombinant insulin (5 μg/mL), penicillin, streptomycin.
Co-culture	Smooth muscle cell growth medium 2 + ECGS (media contains all supplements listed in SMC monoculture, with the addition of bovine hypothalamic extract (12 μg/mL) and heparin (45 μg/mL).

THP-1 monocytes/macrophages were maintained in RPMI media (Thermo Fisher Scientific, Paisley, UK) supplemented with 10% fetal calf serum (FCS; Thermo Fisher Scientific, Inchinnan, UK), L-glutamine (Thermo Fisher Scientific), and Penicillin/Streptomycin (Sigma Aldrich, Dorset, UK). Monocultures of THP-1 were maintained in 75 cm^2^ tissue culture treated vented flasks (Corning) in a 37°C 5% CO^2^ environment. The differentiation of THP-1 monocytes to adherent macrophage-like cells was achieved by stimulating 1 × 10^6^ THP-1 monocytes with 100 ng/mL phorbol 12- myristate 13-acetate (PMA) for 72 h in 6 well tissue culture treated plates (Corning).

### The Triple-Cell Co-culture Methodology

Atherosclerosis co-cultures were maintained in a 37°C and 5% CO^2^ environment. SMCs were seeded at a density of 3–5 × 10^4^ cells/cm^2^ on the underside of Geltrex (Thermo Fisher Scientific) coated 0.4 or 3 μm pore 6 well transwell inserts (Corning). This was achieved by inverting the entire plate containing the insert. The bottom of the plate was then removed leaving the inserts inverted standing on the lid of the plate. SMCs were then seeded in a small volume of SMC monoculture media (200 μl for six well plate) on the bottom of the insert. The bottom of the culture plate was then placed back on top of the inserts; when placing the plate back on top of the inserts the droplet of media will spread to cover the majority of the insert. The SMCs were then allowed to adhere to the transwell membrane for 1 h at 37°C and 5% CO^2^. The plate was then reoriented and 2 mL SMC monoculture media was gently added into the well with 1 mL then added on top of the insert. Inserts remained in culture until SMCs reached confluence, with media changed every 2–3 days. Following this, inserts were transferred to a new six well plate containing fresh co-culture media. ECs in co-culture media were then seeded at confluent density (4 × 10^4^ cells/cm^2^) on the upper surface of the transwell inserts and rested for 24 h. Immune compartments were prepared accordingly to ensure they were ready for use following the 24 h rest period. In our case, THP-1 macrophages were differentiated in separate six well plates as described above; when required for use in the co-culture system THP-1 were stimulated with 100 ng/mL LPS for 2 h to enhance cytokine production (22, 23), washed thoroughly to remove any residual LPS, and wells filled with co-culture media. Co-culture inserts were then transferred into wells containing differentiated THP-1. For all experiments EC+THP-1, SMC+THP-1, EC+SMC, and EC+SMC+THP-1 co-cultures were cultured in co-culture media for the same duration.

### Isolation of Co-culture Cell Layers

ECs were isolated from cultures first by mechanical disruption using a rubber syringe plunger, and carefully removed from the transwell insert and transferred to a suitable vessel using a pipette. Transwells were then transferred to a separate plate containing ice-cold PBS. To remove SMCs, transwells were held at an angle in a fresh recipient well containing ice-cold PBS, SMC layers were mechanically disrupted using a cell scraper and the bottom of the transwell was washed thoroughly to ensure all SMCs were removed into the well. SMCs were then transferred into a suitable vessel. THP-1 cells were isolated last from the bottom of the plates via mechanical disruption with a cell scraper and transferred to a suitable vessel.

### Immunofluorescent Imaging of Co-culture Inserts

Co-culture inserts were fixed in 4% Paraformaldehyde, blocked using Dako serum free protein block and then fluorescently labeled with anti-α smooth muscle cell actin (αSMA) conjugated to FITC (Sigma, Dorset, UK; Cat# F3777) for the identification of SMCs and anti-CD31 conjugated to Alexa-Fluor 594 (Biolegend, London, UK; Cat# 303126) for the identification of ECs. Images were acquired using a Zeiss Cell Observer SD (Zeiss, Jena, Germany) equipped with a Yokagawa CSU-X1 spinning disk system.

### Western Blot

Each cell layer was isolated as described above; proteins were extracted from cells using RIPA lysis buffer (50 mM Tris-HCl, 150 mM NaCl, 0.25% Sodium Deoxycholate, 1 mM EDTA, 1% Nonidet P-40 with protease and phosphatase inhibitors; ThermoFisher Scientific). Samples were resolved on precast 4–20% SDS/PAGE Gels (Expedeon, CA, USA) and transferred to nitrocellulose membranes prior to immunoblotting with antibodies against endothelial nitric oxide synthase (eNOS; R&D Systems, Abingon, UK; Cat# AF950), α-SMA (Agilent, CA, USA; Clone 1A4), and CD11b (R&D systems, Cat# MAB16992). Anti-goat- (Cat# HAF017; R&D systems) and anti-mouse- (Cat# NA931V; GE Healthcare, IL, USA)—HRP secondary antibodies were used as required. Blots were visualized using a Licor C-DiGit (Licor, NE, USA) and Advansta WesternBright ECL (Advansta, CA, USA).

### Quantitative Real-Time Reverse Transcription Polymerase Chain Reaction (qRT-PCR)

Each cell layer was isolated as described above; cells were then centrifuged, and cell pellets resuspended in qiazol lysis reagent (Cat# 79306; Qiagen, Manchester, UK). Total RNA was extracted from ECs and SMCs using the miRNeasy mini kit (Qiagen, Manchester, UK) following manufacturer's instructions. For mRNA analysis, RNA was reverse transcribed using the Taqman® Reverse Transcription Kit (ThermoFisher Scientific) and random hexamer primers according to manufacturer's instructions. qPCR reactions were performed using Taqman® mastermix, primers and probes on a Quantstudio 12K flex real-time PCR system (ThermoFisher Scientific). Each reaction contained 1X TaqMan® Mastermix, 1X cDNA probe (GAPDH: Hs02786624_g1/ NOS3: Hs01574665_m1/VCAM1: Hs01003372_m1/ CDH5: Hs00901465_m1/ PCNA: Hs00427214_g1/ ACTA2: Hs00426835_g1/ CNN: Hs00959434_g1/ TAGLN: Hs01038777_g1 - all ThermoFisher Scientific) and 1.5 μL RT product in a total volume of 10 μL. Real-time PCR was performed at 95°C for 10 min, followed by 40 cycles of 95°C for 15 s and 60°C for 1 min. All PCRs were performed in duplicate wells per plate. The threshold cycle (Ct) was used to determine the relative quantities of each mRNA. Measurements were normalized to the housekeeper UBC (ΔCt) and the inverse log of ΔΔCt gave the relative fold change (denoted as RQ).

### Analysis of Soluble Inflammatory Mediators

Following 24 h of co-culture, supernatants were isolated from double and triple-cell cultures and centrifuged at 10,000 g for 10 min to remove particulates. The remaining supernatant was then assessed for concentrations of E-selectin, granulocyte-macrophage colony-stimulating factor (GM-CSF), interferon-α (IFN-α), IFN-γ, interleukin (IL)-1α, IL-1β, IL-10, IL-12p70, IL-13, IL-17A, IL-4, IL-6, IL-8, IP-10, monocyte chemoattractant protein-1 (MCP-1), macrophage inflammatory protein-1α (MIP-1α), MIP-1β, P-selectin, soluble intercellular adhesion molecule-1 (sICAM-1) and tumor necrosis factor-α (TNF-α), using a 20-plex human cytokine assay according to the manufacturer's instructions (ThermoFisher Scientific) and analyzed on a Bio-plex 200 Luminex system (Bio-Rad Laboratories Ltd). Data was analyzed using Bio-Plex 6.1 software with 5PL curve fitting.

### Statistical Analysis

Data are presented as mean ± standard error of the mean (SEM). One-way ANOVA or repeated measures one-way ANOVA with Tukey's *post-hoc* tests were performed to determine significance between groups; statistical analyses of qPCR data were performed on ΔCt values. Significance was set at *P* < 0.05. All statistical analyses were performed using GraphPad Prism v.8 (GraphPad Software, USA).

## Results and Discussion

It is well recognized that the interplay between ECs, SMCs and the immune system is central to the progression and outcome of cardiovascular disease and atherosclerosis (3, 5, 11). However, *in vitro* mechanistic experiments performed in cell culture often lack this critical element. Consequently, in order to generate *in vitro* data of high relevance to the complexity of human atherosclerosis, the use of vascular-immune co-cultures has been explored. Here we have described the development of a modular co-culture system which facilitates the separation of each cellular compartment in a technically simple manner that avoids the use of cell sorting, which can modify cell properties in an untoward way.

Several models utilizing the co-culture of ECs, SMCs and macrophages have been published (16). Predominantly these models use at least one aspect of direct cellular contact, whereby different cell types are cultured sequentially to provide confluent layers stacked on top of one another (24). Alternatively, some models separate one cellular layer using a transwell membrane system (16, 25). In some cases these direct contact models are highly advanced, for example, Mallone and colleagues developed a spheroid model of atherosclerotic plaque comprising of human myofibroblasts and peripheral blood mononuclear cells (PBMC) (26). The use of PBMCs in this model was highly advantageous, producing spheroid “plaques” with a heterogeneous population of immune cells suitable for investigation. In contrast, Robert and colleagues developed an approach involving the seeding of tubular scaffolds with human myofibroblasts, which were then cultured under flow and subsequently seeded with endothelial cells to produce highly arterial-like *in vitro* vessels (20). The authors demonstrated the potential of this model by briefly exploring the response to atherogenic low-density lipoproteins and macrophage adhesion/transmigration in the cultured vessel. However, whilst both of these models have clear utility, they also have some limitations; both models lack *bona fide* SMCs, whilst the model described by Mallone and colleagues also lacks ECs. Importantly, both approaches would require cell sorting in order to isolate each cell type for classical molecular analyses such as Western Blotting and qRT-PCR.

**Figure 1:**
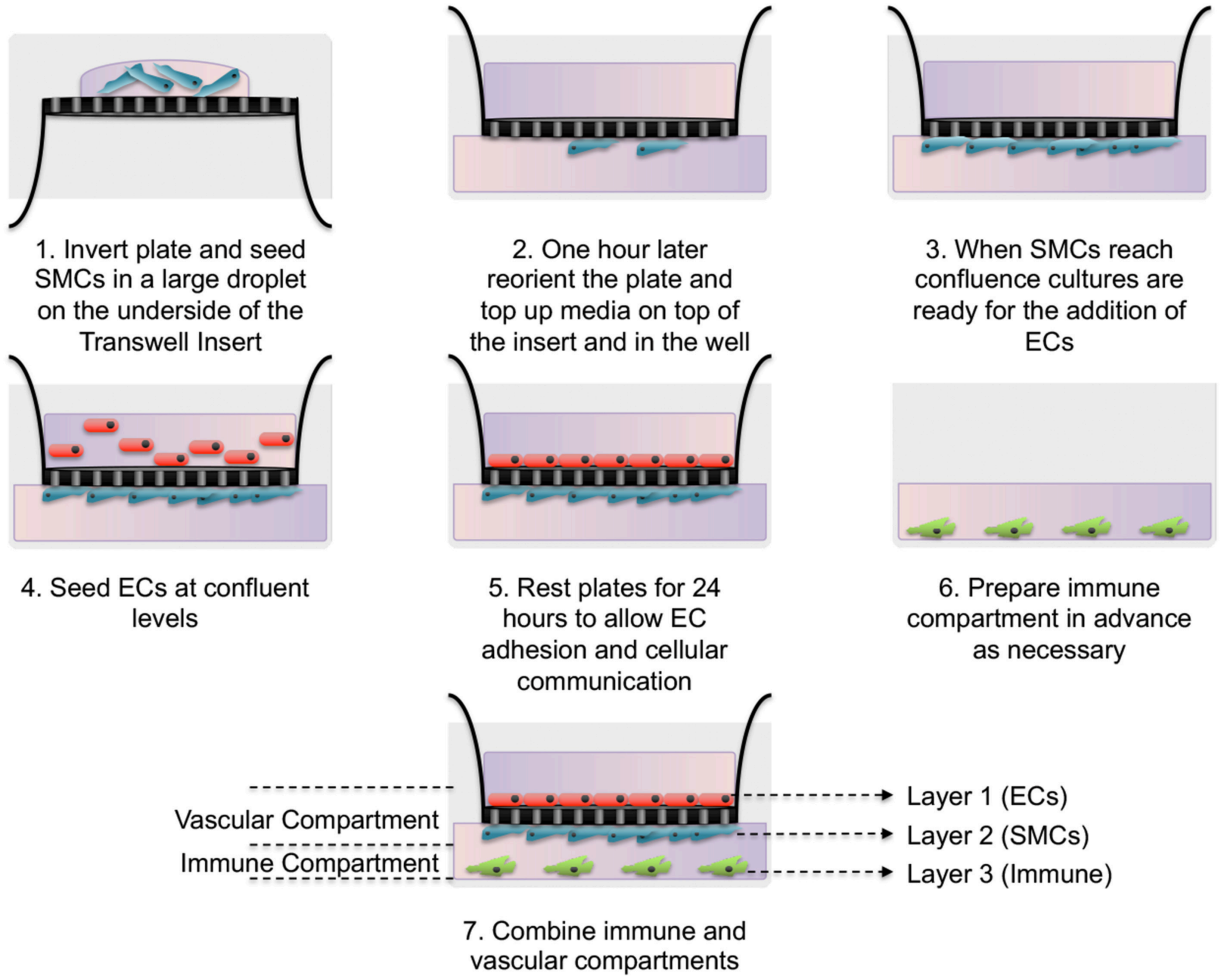
Schematic of triple-cell 2D co-culture methodology. Vascular smooth muscle cells (SMCs) were cultured on the underside of transwell inserts by inverting the entire plate. Once confluent vascular endothelial cells (ECs) were cultured on the upper side. This EC/SMC co-culture was then added to an immune compartment, in this case PMA differentiated THP-1 monocytes.

Here we have developed a triple-cell 2D model of the atheroma which facilitates the interaction of immune cells with a vascular compartment via soluble mediators, whilst allowing the pure isolation of each cellular layer without a need for cell sorting. To achieve our aims, SMCs were seeded on the underside of transwell inserts and cultured until confluent. In parallel, THP-1 monocytes were differentiated into macrophage-like cells using PMA. When SMCs reached confluence ECs were seeded on the upper side of transwell inserts and allowed to adhere for 24 h. Inserts were then transferred into plates containing LPS stimulated adherent macrophage-like THP-1 cells. A diagram of this process is shown in Figure 1.

**Figure 2:**
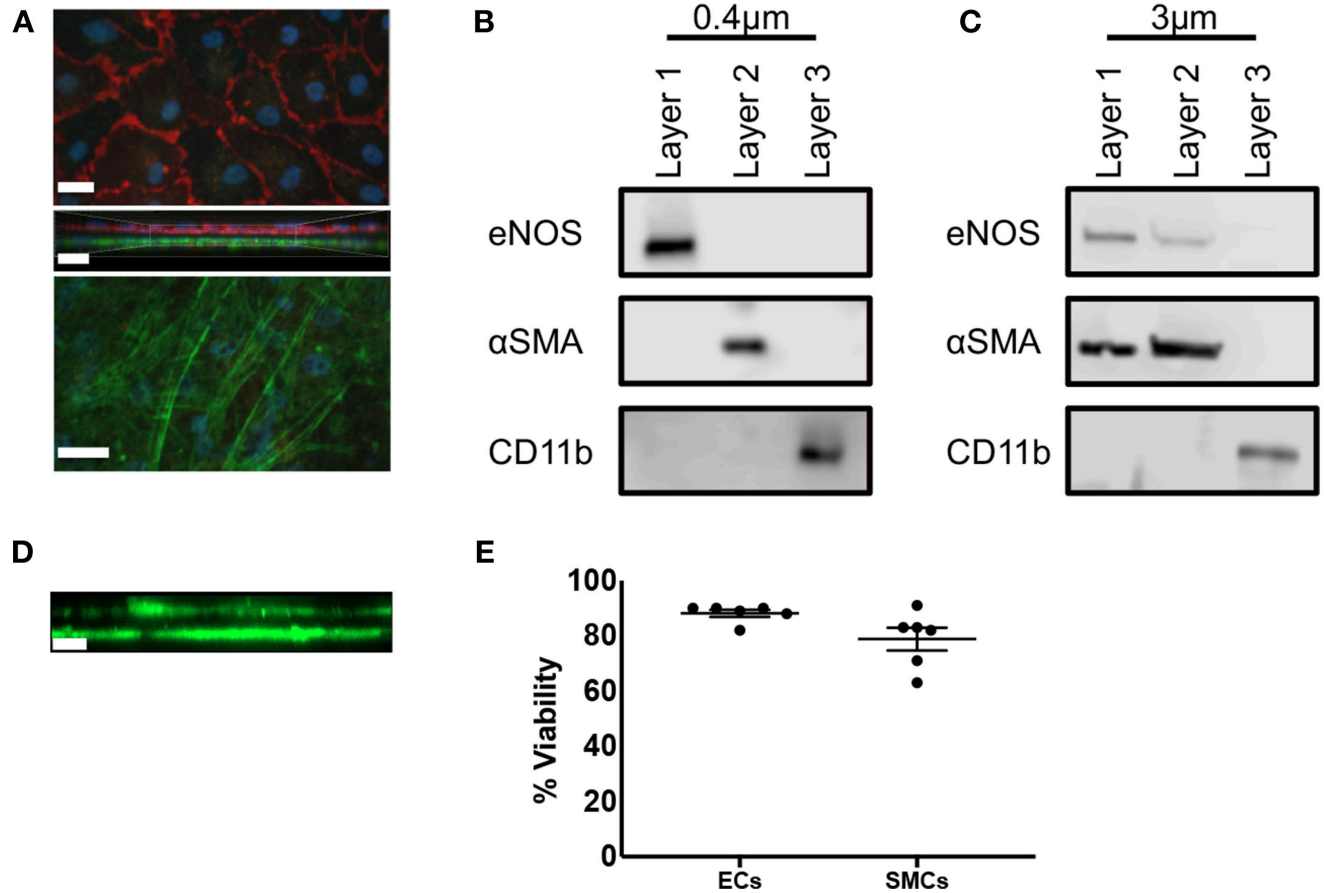
Triple-cell co-culture provides pure cellular niches for macrophages, vascular endothelial and vascular smooth muscle cells. **(A)** Prior to completion of co-cultures via the addition of THP-1 cells, EC/SMC coated transwell inserts were analyzed via immunofluorescent microscopy for CD31 (red) and αSMA (green). Scale Bars = 40 μm. Following completion of EC/SMC/THP-1 co-cultures using both 0.4 μm **(B)** and 3 μm **(C)** transwell inserts, cells were isolated from each cellular niche and analyzed by immunoblotting for eNOS, αSMA, and CD11b. *n* = 3 co-cultures. **(D)** SMC were cultured on the underside of 3 μm transwell inserts and 3 days later analyzed by immunofluorescent microscopy for the expression of αSMA (green). **(E)** ECs or SMCs were cultured on 0.4 μm inserts until confluent. They were then mechanically disrupted and analyzed for viability using trypan blue exclusion. *n* = 6 cultures. Scale Bar = 10 μm.

We first carried out immunofluorescence staining of transwell inserts seeded with SMCs and ECs prior to transfer into THP-1 containing plates. CD31 and αSMA staining of 0.4 μm pored transwell inserts identified confluent populations of SMCs and ECs separated by the thin transwell membrane (Figure 2A). We next confirmed that each cell population was free from contamination by the other cell types. ECs were isolated from confluent EC/SMC/THP-1 co-cultures first by mechanical disruption using a rubber syringe plunger, and carefully removed from the transwell insert and transferred to a suitable vessel. Transwells were then transferred to a separate plate containing ice-cold PBS filled wells. To remove SMCs, transwells were held at an angle in the recipient well, SMC layers were mechanically disrupted using a cell scraper and the bottom of the transwell washed thoroughly to ensure all SMCs were removed. THP-1 cells adhering to the base of the coculture plates were also isolated via mechanical disruption with a cell scraper. Mechanical disruption is commonly used to isolate macrophages, but not SMCs and ECs. To ensure this was not an issue for these cell types we carried out analysis of viability using trypan blue staining. Here, we found viabilities of ~90% and ~80% for ECs and SMCs, respectively, confirming this method of isolation did not significantly compromise the isolated cells (Figure 2E). Once each layer was isolated Western Blot analysis was conducted for eNOS, αSMA, and CD11b, used to discriminate ECs, SMCs, and THP-1, respectively. Co-cultures using 0.4 μm pored inserts yielded pure cell populations, with no evident contamination between layers from adjacent cell types and confirming the ability to individually isolate each culture layer (Figure 2B). Contrastingly, the use of 3 μm inserts allowed for the migration of SMCs through into the EC layer and vice versa (Figure 2C). We confirmed this observation by seeding SMCs on the underside of 3 μm transwell inserts and 3 days later carried out immunofluorescent microscopy for αSMA to identify if SMCs could migrate from the bottom to the top of the insert. As expected, we found SMCs on both sides of the transwell membrane (Figure 2D). Consequently, if mixed EC/SMC populations were desired to facilitate direct cellular contact this would be possible using 3 μm inserts.

**Figure 3:**
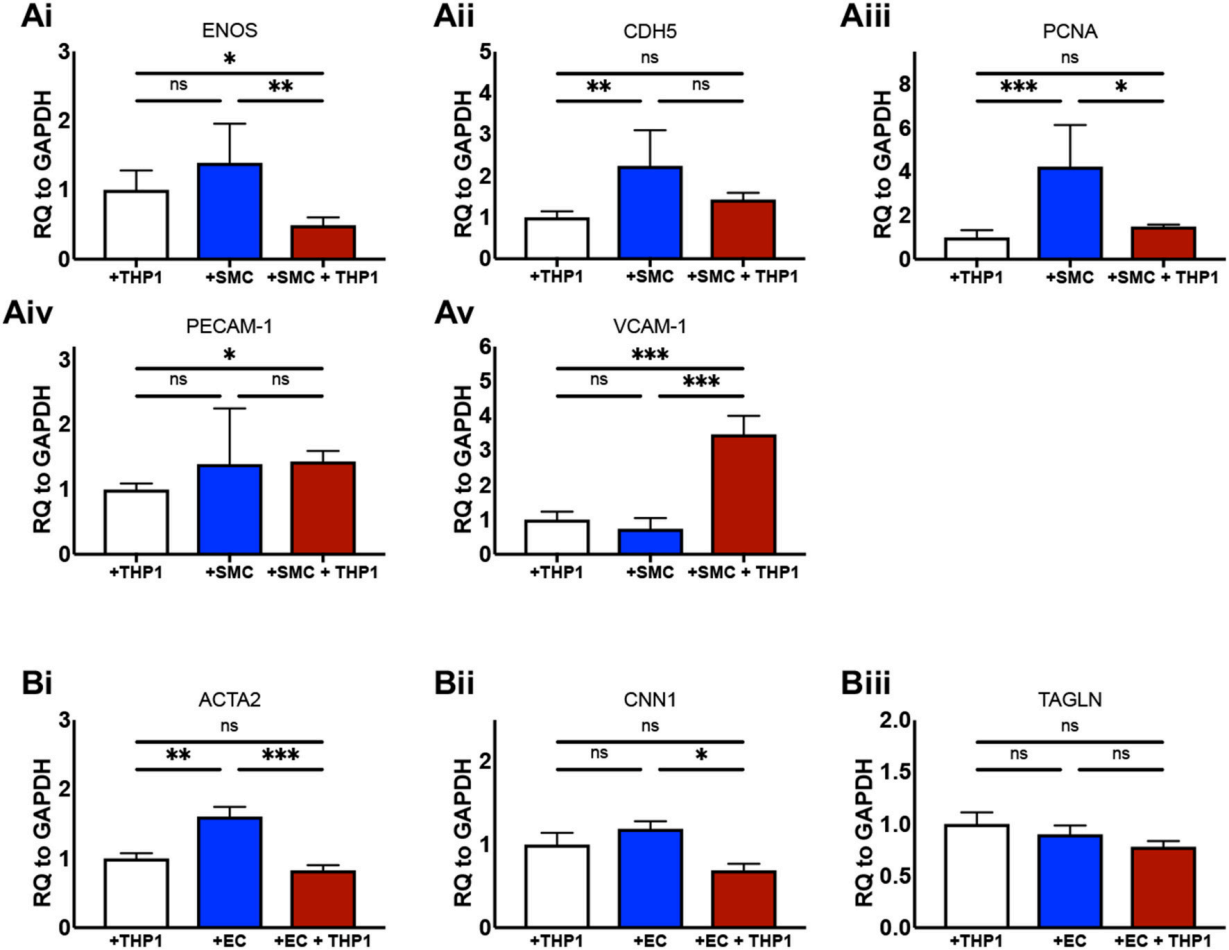
Altered cellular phenotype by macrophage, vascular endothelial and vascular smooth muscle cell interaction. ECs were co-cultured with THP-1, SMCs, or both THP-1 and SMCs simultaneously. Twenty-Four hours following co-culture each cell type was isolated from culture and analyzed via RT-qPCR for the expression of **(Ai)** *eNOS*, **(Aii)** *CDH5*, **(Aiii)** *PCNA*, **(Aiv)** *PECAM-1*, and **(Av)** *VCAM-1*. SMCs were co-cultured with THP-1, ECs, or both THP-1 and ECs simultaneously, and analyzed for the expression of **(Bi)** *ACTA2*, **(Bii)** *CNN1*, and **(Biii)** *TAGLN*. Results were analyzed by one-way ANOVA with Tukey's multiple comparisons test. ^*^P < 0.05, ^**^P < 0.01, ^***^P < 0.001. *n* = 4–6 co-cultures.

We next aimed to confirm that the culture of ECs and SMCs together with an “immune” compartment in 2D produced biologically relevant changes to the phenotype of the cells in our model in comparison with equivalent double-cell 2D cultures. It is known that the culture of ECs with SMCs in non-inflammatory conditions increases *eNOS* expression (27). We found that ECs from EC/SMC co-cultures displayed a trend toward increased *eNOS* expression in comparison to EC/THP-1 co-cultures, though this did not reach significance (Figure 3Ai). Interestingly, we observed that the co-culture of ECs, SMCs, and THP-1 together induced a significant repression of EC *eNOS*, suggesting that triple-cell co-culture enhances the impact of inflammation on EC phenotype. We next investigated the expression of *CDH5; CDH5* encodes VE-cadherin, which contributes to the integrity of intercellular junctions and endothelial permeability. We found that triple-cell co-cultured ECs had *CDH5* levels similar to those of ECs cultured with THP-1 alone (Figure 3Aii). However, we also noted that EC/THP-1 co-cultures displayed significantly reduced *CDH5* when compared with EC/SMC cultures. ECs from triple-cell cultures also showed a trend toward reduced *CDH5* expression in comparison with EC/SMC cultures, though this did not reach significance. Therefore, our results suggest that EC/SMC co-cultures have increased EC junction integrity in comparison with cultures containing an inflammatory compartment, as one might expect. We found expression of the proliferation marker *PCNA* to be reduced in ECs exposed to THP-1, irrespective of the presence of SMCs (Figure 3Aiii). Furthermore, we identified a small but significant increase in platelet endothelial cell adhesion molecule 1 (*PECAM-1*) in EC/THP-1 vs EC/SMC/THP-1 (Figure 3Aiv) alongside a striking increase in vascular cell adhesion molecular 1 (*VCAM-1*) in triple-cell cultured ECs when compared to both double-cell 2D conditions (Figure 3Av). The increase in VCAM-1 is particularly interesting. VCAM-1 is an adhesion molecule fundamental to the recruitment of immune cells and has been identified as a potentially useful biomarker for molecular imaging in cardiovascular disease (28). Its significant upregulation by the presence of SMCs and THP-1, but not either cell type alone, highlights a role for the multi-directional communication between ECs, SMCs and macrophages in modulating adhesion molecule expression. In addition to analyzing ECs, we also investigated the expression of smooth muscle α-actin (*ACTA2*), calponin 1 (*CNN1*), and transgelin (*TAGLN*) in SMCs from SMC/THP-1, EC/SMC, and EC/SMC/THP-1 co-cultures. Expression of all three markers is associated with a contractile SMC phenotype (29). No differences in *TAGLN* were observed between co-cultures, however we did identify a decrease in *ACTA2* expression in the SMC/THP-1 and EC/SMC/THP-1 co-cultures when compared with EC/SMC co-cultures (Figure 3B). Furthermore, we identified a significant decrease in *CNN1* expression between the EC/SMC/THP-1, but not SMC/THP-1, when comparing with EC/SMC co-cultures (Figure 3B). This data suggests a less contractile smooth muscle phenotype in cultures containing THP-1, consistent with previous studies showing that acquisition of a pro-inflammatory SMC phenotype is associated with downregulation of SMC contractile genes (30). Whist further analysis would be required to clarify the full phenotypic impact of the triple-cell co-culture on SMCs, the data again highlights the possibility to isolate individual cell types from this culture system and carry out further analyses. The distribution of ΔCT values for all data in Figure 3 is presented in Supplemental Figure 1.

**Figure 4:**
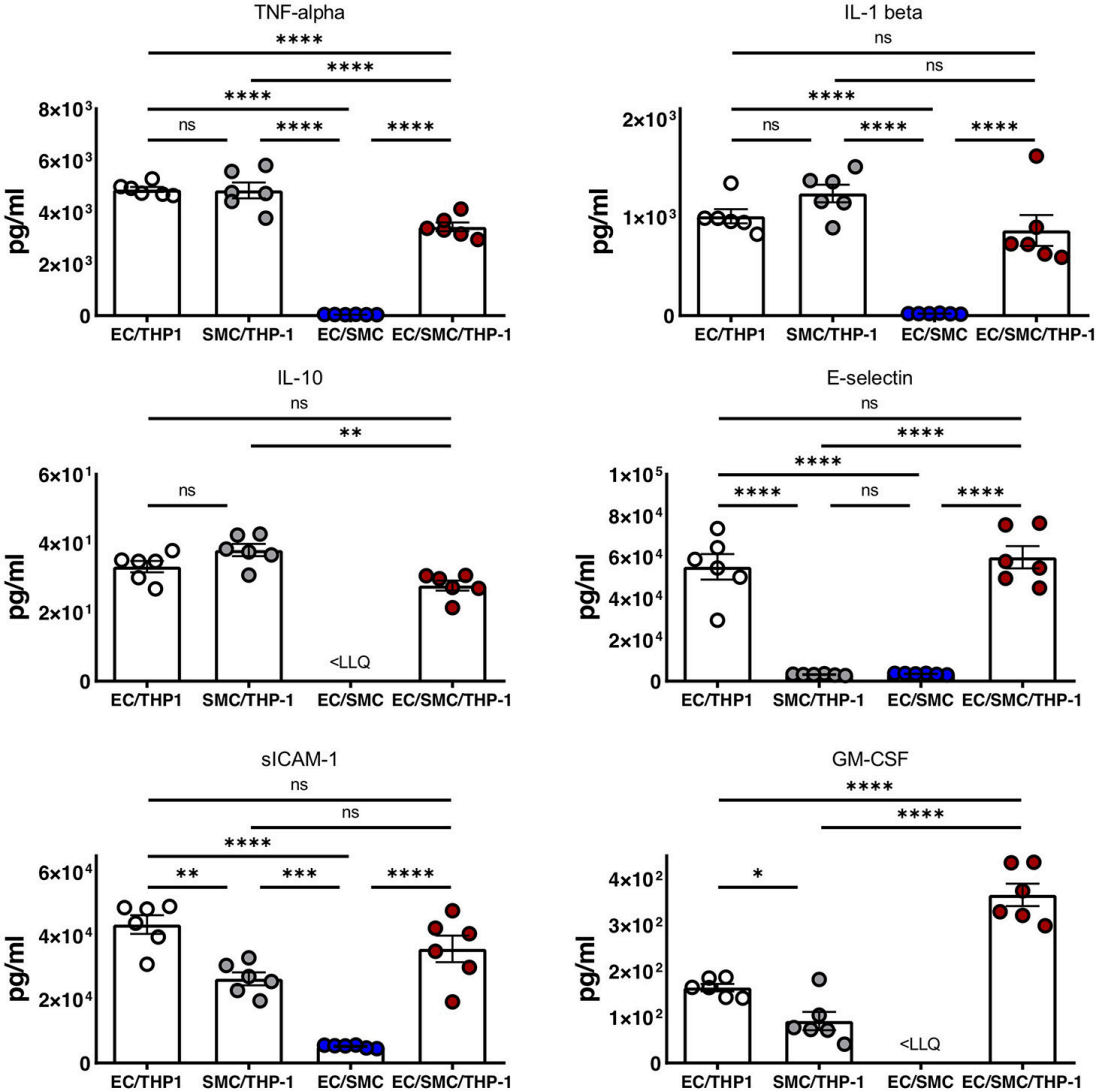
Soluble mediator profile of macrophage, vascular endothelial and vascular smooth muscle cell co-cultures. EC/THP-1, SMC/THP-1, EC/SMC, and EC/SMC/THP-1 co-cultures were prepared. Twenty-four hours later co-culture supernatants were collected and analyzed for the presence of TNF-α, IL-1β, IL-10, E-selectin, sICAM-1, and GM-CSF by Luminex. Results were analyzed by one-way ANOVA with Tukey's multiple comparisons test. ^*^P < 0.05, ^**^P < 0.01, ^***^P < 0.001, ^****^P < 0.0001. *n* = 6 co-cultures. <LLQ, less than Lower Limit of Quantification.

Finally, we utilized Luminex to quantify the expression of soluble mediators from the supernatants from EC/THP-1, SMC/THP-1, EC/SMC, and EC/SMC/THP-1 co-cultures. In this comparison we present data from the analysis of TNF-α, IL-1β, IL-10, E-selectin, sICAM-1, and GM-CSF (Figure 4). We found significant differences in these molecules in between at least two co-culture models. We identified that E-selectin was only detectable when co-cultures contain both ECs and THP-1 but was not dependent on the presence of SMCs. Furthermore, we found that the production of TNF-α, IL-1β, IL-10, sICAM-1, and GM-CSF appeared reliant on the presence of the macrophage compartment, and thus an inflammatory stimulus. Whilst the production of these molecules was dependent on THP-1, we did find significant differences between our triple-cell and the comparative THP-1 containing double cell co-cultures. Most interestingly, we found our triple-cell culture had a mildly, but significantly, blunted TNF-alpha response in comparison with EC/THP-1 and SMC/THP-1 cultures, and significantly increased GM-CSF production. This data again highlights the importance of utilizing more advanced co-culture methodologies, such as our triple-cell 2D model, over similar double cell cultures.

In summary, the observation that EC/SMC/THP-1 triple-cell 2D cultures create a significantly different phenotype in respect to both soluble mediators and cell surface molecules, when compared to double-cell cultures, underscores the utility and potential of our approach for the study of key vascular-inflammatory mechanisms in the context of multi-cellular communication.

## Conclusions

We have developed a co-culture model for the study of the immune-vascular interplay in atherosclerosis consisting of ECs, SMCs and an inflammatory compartment. In order to validate this model, we have demonstrated that the triple-cell 2D culture of ECs, SMCs, and THP-1 macrophage-like cells significantly alters the phenotype of ECs and SMCs within this culture in comparison with EC/SMC, EC/THP-1, or SMC/THP-1 double-cell cultures. Our results are indicative of the multi-directional communication between highly plastic vascular stromal cells and the immune system, which is now well recognized in the field, and highlight the applicability of this model for investigating cumulative immune-vascular responses *in vitro*. In order to increase the relevance of *in vitro* studies to human disease, where possible, we should utilize systems more accurately reflecting the cellular complexity of atherosclerosis. One limitation that must be noted is the lack of cell contact, which would be experienced by these cells in the vessel. However, even in the absence of these interactions, clear phenotypic differences in ECs and SMCs from triple vs. double-cell 2D cultures were observed. In addition, when using 0.4 μm pored inserts it would not be possible to study transmigration of immune cells thorough both the endothelial and smooth muscle layer, though studying cell adhesion would be possible. Whilst the model presented utilized THP-1 cells as the immune compartment, the use of PBMCs, or other immune cell populations, would be a highly useful extrapolation of our data. Ultimately, our aim was not to develop one system, but to provide a framework which could be adapted for specific immune and vascular cells of interest.

The main utility of this model is the ability to isolate each cellular compartment without cell sorting in order to carry out classical molecular analyses similar to those conducted in this study. We would suggest that this triple-cell 2D model would be highly useful for studying the impact of key inflammatory mediators and atherogenic stimuli at the molecular level whilst retaining the important biological influence of immune and vascular cell communication in a simple and robust manner.

## Author Contributions

JN designed and performed experiments and wrote the manuscript. GG designed and performed experiments. NM performed experiments. TG and PG were involved in study design and revised the manuscript. AB performed experiments and wrote the manuscript. PM designed experiments and wrote the manuscript. All authors have read and approved the final manuscript.

### Conflict of Interest Statement

The authors declare that the research was conducted in the absence of any commercial or financial relationships that could be construed as a potential conflict of interest.
