# Supplementary material for: A Novel Triple-Cell Two-Dimensional Model to Study Immune-Vascular Interplay in Atherosclerosis
Source: Front Immunol. 2019 Apr 24;10:849. doi: 10.3389/fimmu.2019.00849 (PMC6491724; doi:10.3389/fimmu.2019.00849)
Supplement: Supplementary file 1 [file Data_Sheet_1.docx]

**Supplemental Figure 1.** Distribution of 𝛥CT values for data shown in Figure 3. 𝛥CT values are shown to demonstrate the distribution of the data for experiments exploring altered cellular phenotype as a result of macrophage, vascular endothelial and vascular smooth muscle cell interaction. ECs were co-cultured with THP-1, SMCs or both THP-1 and SMCs simultaneously. 24 hours following co-culture each cell type was isolated from culture and analysed via RT-qPCR. ECs were analysed for the expression of (**Ai**) *eNOS*, (**Aii**) *CDH5*, (**Aiii**) *PCNA*, (**Aiv**) *PECAM-1* and (**Av**) *VCAM-1*. SMCs were co-cultured with THP-1, ECs or both THP-1 and ECs simultaneously and analysed for the expression of (**Bi**) *ACTA2*, (**Bii**) *CNN1* and (**Biii**) *TAGLN*. n=4-6 cultures.
